# Adipocyte Fatty Acid-Binding Protein, Cardiovascular Diseases and Mortality

**DOI:** 10.3389/fimmu.2021.589206

**Published:** 2021-03-19

**Authors:** Chi-Ho Lee, David T. W. Lui, Karen S. L. Lam

**Affiliations:** ^1^Department of Medicine, University of Hong Kong, Hong Kong, Hong Kong; ^2^State Key Laboratory of Pharmaceutical Biotechnology, University of Hong Kong, Hong Kong, Hong Kong

**Keywords:** cardiovascular disease, adipocyte fatty acid-binding protein, mortality, inflammation, adipokine

## Abstract

It has been increasingly recognized that inflammation plays an important role in the pathogenesis of cardiovascular disease (CVD). In obesity, adipose tissue inflammation, especially in the visceral fat depots, contributes to systemic inflammation and promotes the development of atherosclerosis. Adipocyte fatty acid-binding protein (AFABP), a lipid chaperone abundantly secreted from the adipocytes and macrophages, is one of the key players mediating this adipose-vascular cross-talk, in part *via* its interaction with c-Jun NH2-terminal kinase (JNK) and activator protein-1 (AP-1) to form a positive feedback loop, and perpetuate inflammatory responses. In mice, selective JNK inactivation in the adipose tissue significantly reduced the expression of AFABP in their adipose tissue, as well as circulating AFABP levels. Importantly, fat transplant experiments showed that adipose-specific JNK inactivation in the visceral fat was sufficient to protect mice with apoE deficiency from atherosclerosis, with the beneficial effects attenuated by the continuous infusion of recombinant AFABP, supporting the role of AFABP as the link between visceral fat inflammation and atherosclerosis. In humans, raised circulating AFABP levels are associated with incident metabolic syndrome, type 2 diabetes and CVD, as well as non-alcoholic steatohepatitis, diabetic nephropathy and adverse renal outcomes, all being conditions closely related to inflammation and enhanced CV mortality. Collectively, these clinical data have provided support to AFABP as an important adipokine linking obesity, inflammation and CVD. This review will discuss recent findings on the role of AFABP in CVD and mortality, the possible underlying mechanisms, and pharmacological inhibition of AFABP as a potential strategy to combat CVD.

## Introduction

Obesity is a global health problem. Based on the data from the World Health Organization (WHO), in 2016, more than 1.9 billion adults aged 18 years or above were overweight, and among them, 650 million were obese (1). In a pooled analysis of 19.2 million participants, the age-standardized prevalence of obesity has tripled in men and doubled in women over the last four decades. If these trends continue, around 1 in 5 of the global population will become obese by year 2025 (2).

Obesity leads to increased risks of type 2 diabetes (3, 4), non-alcoholic fatty liver disease (NAFLD) (5), cardiovascular disease (CVD) (6), cancer (7), and mortality. Indeed, high body mass index (BMI) has become one of the top five leading causes of all-cause mortality and disability-adjusted life-years (8). In 2015, high BMI contributed to 7.1% of global deaths. Strikingly, CVD accounted for two-thirds of these deaths and more than half of disability-adjusted life-years related to high BMI (9). Recently, in a Mendelian randomization (MR) study involving more than 360,000 participants from the UK Biobank, each genetically instrumented increase in BMI of 1 kg/m^2^ was associated with a significantly higher risk of most cardiovascular outcomes including hypertension, atrial fibrillation, coronary heart disease (CHD), heart failure and peripheral vascular disease (PVD) (10). Genetically predicted fat mass index was associated with an even broader list of cardiovascular outcomes including ischemic stroke. These findings corroborated with another large MR study which demonstrated the causal effects of adiposity on CVD (11). Taken together, both observational and MR studies provided strong epidemiological evidence that obesity, in particular central adiposity, is closely linked with CVD and cardiovascular mortality.

Inflammation, on the other hand, is an established important risk factor of CVD and cardiovascular mortality (12). Previous observational studies had demonstrated that markers of inflammation such as C-reactive protein (CRP) and tumor necrosis factor alpha (TNF-α) receptor 1 were independent prognostic markers of adverse cardiovascular outcomes among individuals with and without prevalent CVD (13, 14). Recently, the use of Canakinumab, an anti-inflammatory monoclonal antibody targeting interleukin-1, was also shown in a randomized controlled trial to significantly reduce the incidence of non-fatal myocardial infarction, non-fatal stroke and cardiovascular death, confirming that inflammation plays a crucial role in the pathogenesis of CVD (15). Obesity is a state of chronic low-grade systemic inflammation, which is induced by a cascade of cellular events that occur in the dysfunctional adipose tissue, and perpetuated by dysregulated secretion of adipokines through their local and systemic actions (16). This review will focus on adipocyte fatty acid-binding protein (AFABP) and present the recent data on its role as an important adipokine linking obesity, inflammation and CVD.

## AFABP Expression and Secretion

AFABP is a major cytosolic protein of the mature adipocytes (17). As a fatty acid binding protein, it acts as a lipid chaperone that facilitates the trafficking of non-esterified fatty acids throughout cellular compartments such as peroxisome, endoplasmic reticulum (ER), mitochondria and nucleus (18). AFABP also regulates lipid storage and oxidation, and is involved in lipolysis though its interaction with the hormone-sensitive lipase (HSL) and a co-activator of adipose triglyceride lipase (ATGL) (19, 20). The expression of AFABP in adipocytes is induced during adipocyte differentiation, and is transcriptionally activated by fatty acids, glucocorticoids, cyclic adenosine monophosphate (cAMP), and peroxisome proliferator-activated receptor gamma (PPARγ) agonists (21–23).

Studies in recent years have shown that AFABP is secreted from the adipocytes, and circulates in the blood stream in both mice and humans (24) (25). However, since it lacks a signal peptide sequence for classical secretory pathway (25), it has recently been reported that AFABP is secreted unconventionally *via* endosomes and secretory lysosomes in response to lipolytic and fasting related signals, such as adrenergic signaling, beta agonists, branched-chain amino acids and glycerol (25, 26), and the involvement of sirtuin-1 activation has been implicated (27). While it is also expressed in the macrophages (28) and endothelial cells (29), *in vivo* data suggest that the adipocyte is the predominant contributor to circulating AFABP levels (25).

## AFABP in Relation to Adipose Tissue Inflammation and Insulin Resistance in Obesity

AFABP secretion is dysregulated in obesity, with raised circulating AFABP concentrations being found in obese individuals (24). With chronic nutrient excess, pathological expansion of the adipose tissue causes several maladaptive changes especially in the visceral fat depots. Hypertrophic adipocytes undergo high rates of spontaneous lipolysis (30), which increases free fatty acid (FFA) efflux and stimulates AFABP release. Lipo-toxicity ensues as lipid intermediates such as ceramides and diacylglycerols accumulate. Moreover, adipocyte hypoxia and cell death develop as a consequence of its continuous expansion despite relative under-perfusion and increased mechanical stress (31), and hypoxia is another known stimulus for AFABP release from adipocytes (32). On the other hand, AFABP (33), as a lipid chaperone, has been implicated in ER stress in response to lipotoxic signals, leading to activation of stress kinases such as nuclear factor kappa B (NFκB) and c-Jun NH2-terminal kinase (JNK) (34), enhancing adipocyte insulin resistance that potentiates lipolysis and lipotoxicity. Adipocyte insulin resistance also augments the secretion of pro-inflammatory cytokines including the chemokine monocyte chemoattractant protein 1 (MCP1) (35), which stimulates the recruitment of macrophages into the adipose tissue (36). Furthermore, it induces a phenotypic switch in the macrophages from the anti-inflammatory M2 polarized state to the pro-inflammatory phenotype typical of M1 classical inflammation in metabolically-activated macrophages (MMe) (37, 38).

Both innate and adaptive immunity are activated in obesity. In addition to macrophage infiltration, adaptive immune cells including CD4+ T helper (Th1) cells, CD8+ T cells and B cells also accumulate in the visceral adipose tissue (39). Transient enhancement of AFABP expression has been reported in murine splenic lymphocytes after dexamethasone administration (40). However, among the major human leucocyte subsets, the expression of AFABP is largely restricted to the macrophages and myeloid dendritic cells (DC) (41). Specifically, owing to its high expression in the macrophages (28), AFABP is more closely linked with the innate immune cells. It has been shown that AFABP perpetuates lipopolysaccharide (LPS)-induced inflammatory responses in macrophages through its interaction with JNK and activator protein-1 (AP-1) forming a positive feedback loop. Upon stimulation by LPS *via* toll like receptor 4 (TLR4), JNK is activated, leading to the induction of c-Jun phosphorylation and its recruitment to a highly conserved AP-1 consensus binding motif located within the AFABP gene promoter. As a result, AFABP gene transcription is upregulated, which further potentiates LPS-induced JNK phosphorylation, activation of AP-1 complex and amplification of pro-inflammatory responses in the macrophages (42). Nonetheless, AFABP can also affect adaptive immunity through the modulation of DC responses. NFκB activation is impaired in AFABP deficient DCs, which exhibit reduced DC function in T cell priming and cytokine production (41). Recently, AFABP was also found to be upregulated in a subpopulation of tissue-resident memory CD8+ T cells which have high requirement for fatty acid metabolism. Importantly, the lack of AFABP in these cells could negatively impact their survival and hence attenuate their function in protective immunity (43). In a viral infection model, mice with genetic deficiency of AFABP had decreased interferon gamma production and increased viral load (41). However, in a rodent model of sepsis, pharmacological inhibition of AFABP in fact was demonstrated to be beneficial, with attenuation of sepsis-triggered inflammatory responses, reduced hepatic and pulmonary tissue injury, as well as improved survival (44). Taken together, these studies highlight the close and complex relationship between AFABP and cellular immunity.

**Figure 1:**
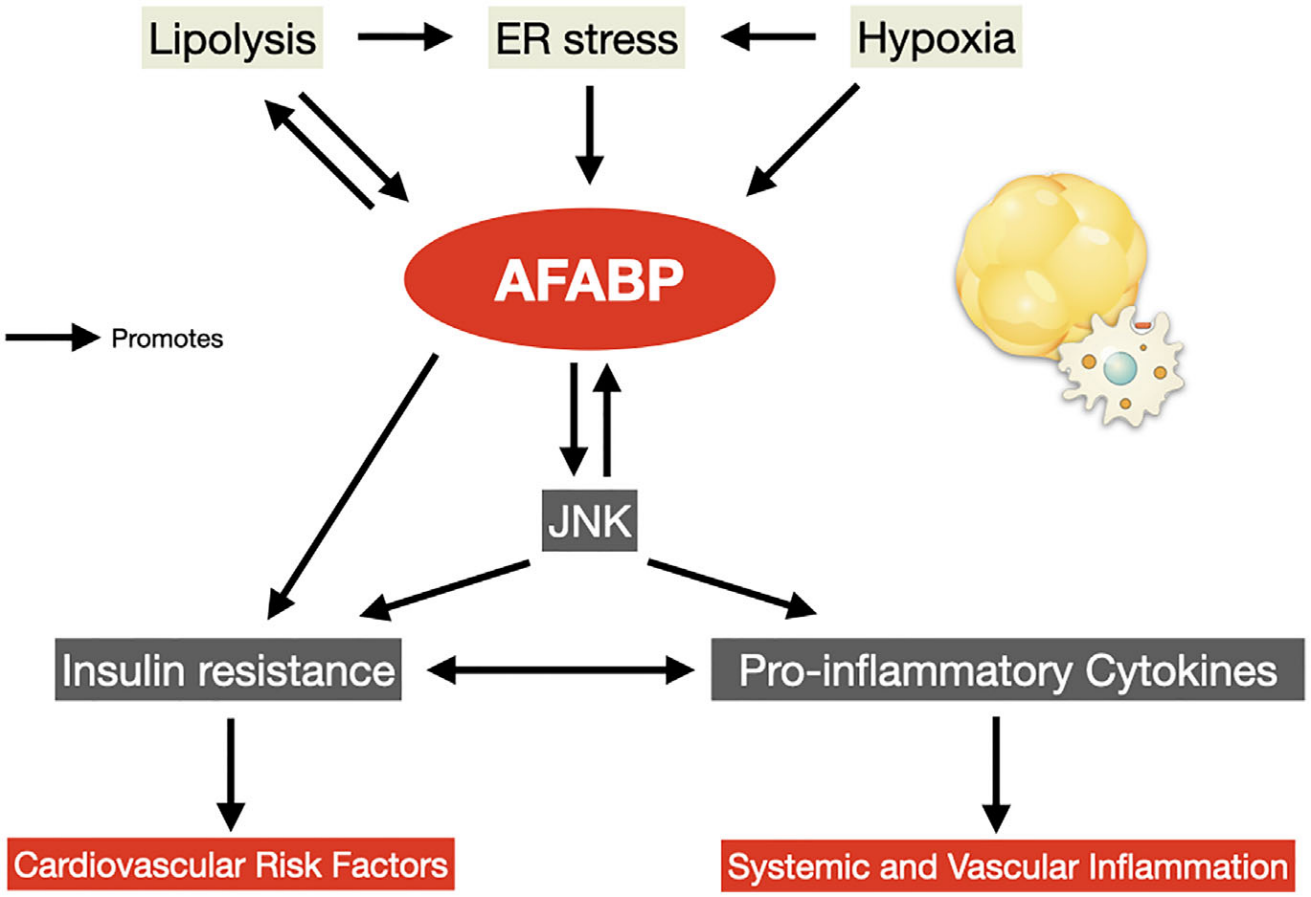
AFABP in the vicious cycle of adipose tissue insulin resistance and inflammation. AFABP, adipocyte fatty acid-binding protein; ER, endoplasmic reticulum; JNK, c-Jun NH2-terminal kinase.

In the adipose tissue, infiltration of these immune cells drives further release of pro-inflammatory adipokines including TNF-α, interleukin-6 (IL-6) and AFABP, and reduces the secretion of the anti-inflammatory adipokine adiponectin. Increased AFABP secretion induces further lipolysis and inflammation in the adipocytes *via* the p38/mitogen-activated protein kinase (MAPK) pathway (45), and contributes to this vicious cycle of adipose tissue insulin resistance and inflammation (46) (**Figure 1**). Whole-body insulin sensitivity was ultimately impaired, accompanied by a chronic state of subclinical systemic inflammation, and the development of an array of obesity-related complications including CVD and cardiovascular mortality (**Table 1**).

**Table 1:** Associations of AFABP with cardiometabolic conditions.

	Circulating AFABP level	Potential mechanistic actions	References
Type 2 diabetes	Predicts the development of type 2 diabetes	Increases free fatty acid efflux Reduces glucose utilization in muscles Increases hepatic expression of gluconeogenic enzymes	(25, 47–51)
Hypertension	Correlates positively with blood pressure	Increases endothelial dysfunction Worsens insulin sensitivity	(47, 48, 50, 52)
Dyslipidemia	Correlates positively with low-density lipoprotein cholesterol Correlates negatively with high-density lipoprotein cholesterol	Increases free fatty acid efflux Negative effects on lipid metabolism Worsens insulin sensitivity	(47, 48, 50)
Coronary heart disease	Predicts the development of cardiovascular diseases Associates with coronary calcium score in patients with type 2 diabetes Associates with the coronary plaque burden in patients with coronary heart disease	Promotes atherosclerosis development: Alters lipid metabolism in macrophages and facilitates foam cell formation Promotes saturated fatty acid-induced ceramide production in macrophages Mediates toxic lipids-induced endoplasmic reticulum stress in macrophages Increases adipose tissue and systemic inflammation	(33, 53–60)
Stroke	Associates with the presence of carotid atherosclerosis Correlates positively with the vulnerable carotid plaque phenotype Doubles the risk of incident adverse cardiovascular events including cardiovascular mortality, non-fatal myocardial infarction and non-fatal stroke. Predicts poor functional outcome and mortality from ischemic stroke	Promotes atherosclerosis development (as above) Enhances the production of matrix metalloproteinases-9 which degrade the tight junction proteins in the blood brain barrier, leading to cerebral edema, increased neuro-inflammation and poor neurological outcomes	(61–68)
Heart failure	Correlates positively with circulating levels of N-terminal fragment of pro-B-type natriuretic peptide Associates with the presence of left ventricular systolic and/or diastolic dysfunction Associates with increasing severity of clinical heart failure Predicts incident heart failure among older individuals	Negative inotropic effect on cardiomyocytes Reduces phosphorylation of endothelial nitric oxide synthase in acute myocardial ischemia/reperfusion injury Increases oxidative stress and cardiac inflammation Increases cardiac hypertrophy and fibrosis	(52, 69–75)
Cardiovascular mortality	Associates with both short- and long-term cardiovascular morbidity and mortality in patients with established coronary heart disease Predicts cardiovascular deaths in patients with type 2 diabetes	See above	(76–80)

## AFABP and Cardiovascular Risk Factors

The detrimental role of AFABP on the development of CVD begins with its effects on traditional cardiovascular risk factors in addition to excess adiposity. AFABP-deficient mice displayed improved glycemia, insulin sensitivity and lipid metabolism in both dietary and genetically induced obesity (47, 48), secondary to a reduced FFA efflux and increased glucose utilization in muscles (49). Moreover, AFABP increases the hepatic expression of gluconeogenic enzymes phosphoenolpyruvate carboxylase 1 (*Pck1*) and glucose-6-phosphatase (*G6pc*), leading to enhanced hepatic glucose production and impaired glucose metabolism (25).

In humans, circulating AFABP concentrations also correlate positively with adverse cardiometabolic risk factors including age, obesity indices, hypertension, homeostatic model of insulin resistance (HOMA-IR), low-density lipoprotein cholesterol (LDL-C), and negatively with high-density lipoprotein cholesterol (HDL-C) (50). Moreover, high circulating AFABP concentrations predicted incident metabolic syndrome and type 2 diabetes, both of which are associated with increased risks of CVD and mortality (50, 51).

## AFABP and Atherosclerosis

AFABP promotes atherosclerosis, the central event in the pathogenesis of CVD (81). Bone marrow transplant experiments revealed that macrophage-specific AFABP deficiency reduced atherosclerotic lesions in mice with apolipoprotein E (ApoE) deficiency, to a similar extent as those with whole body AFABP deficiency, suggesting that much of the pro-atherogenic effects of AFABP are specific to its actions in macrophages (28). The expression of AFABP in macrophages can be upregulated in response to oxidized LDL (oxLDL) and LPS (82, 83), which are both increased in obesity (84, 85). On the other hand, metformin has been shown to inhibit AFABP expression in macrophages (86). AFABP alters lipid metabolism in macrophages and facilitates the formation of foam cell enriched with cholesterol and triglyceride (53, 54). AFABP also promotes macrophage cell death through saturated fatty acid-induced ceramide production (55). Moreover, AFABP has been shown as an obligatory mediator of toxic lipids-induced ER stress in macrophages, through inhibiting liver X receptor alpha (LXRα) to reduce macrophage *de novo* fatty acid synthesis which confers resistance to ER stress (33), as well as impairing macrophage autophagy by attenuation of Janus Kinase 2 (JAK2) activity (87). The elevated ER stress potentiates JNK activation and further exacerbates inflammation.

However, there was recent evidence suggesting that the negative impact of AFABP on atherosclerosis was not exclusively due to its action in the macrophages. In mice, selective JNK inactivation in the adipose tissue significantly reduced both the expression of AFABP in their adipose tissue, as well as circulating AFABP levels. Importantly, fat transplant experiments showed that adipose-specific JNK inactivation in the visceral fat was sufficient to protect mice with apolipoprotein E (ApoE) deficiency from atherosclerosis, with the beneficial effects attenuated by the continuous infusion of recombinant AFABP, supporting the participation of adipocyte-derived AFABP as a link between visceral fat inflammation and atherosclerosis (56).

In humans, elevated baseline AFABP concentration predicted incident CVD over a median follow-up of around 10 years in a community-based cohort (57). Moreover, high circulating AFABP concentration was associated with coronary calcium score in patients with type 2 diabetes (58), as well as the coronary plaque burden in patients with coronary heart disease (59). In keeping with observations from preclinical studies, AFABP was not only expressed in macrophages within atherosclerotic plaques of the coronary arteries in patients with CHD, but also in both macrophages and adipocytes in their epicardial and perivascular fat. *In vitro* studies showed that treatment of human coronary artery smooth muscle and vascular endothelial cells with AFABP augmented palmitic acid-induced inflammation, suggesting that AFABP from epicardial and perivascular fat could also participate in the development of coronary atherosclerosis in a paracrine manner (60). Furthermore, individuals who harbored the single nucleotide polymorphism (SNP) T-87C, which reduced AFABP gene expression in their adipose tissue, was found to have a lower risk of CHD (88).

## AFABP and Stroke

The role of AFABP in the development of stroke is multifaceted. First, high circulating AFABP concentration was associated with the presence of carotid atherosclerosis (61, 62), a predisposing condition for cerebral infarction. In patients with carotid atherosclerosis, AFABP concentrations in their carotid plaques correlated positively with the vulnerable plaque phenotype (63, 64), predicted their disease progression (89), and doubled their risk of incident adverse cardiovascular events including cardiovascular mortality, non-fatal myocardial infarction and non-fatal stroke (64). Moreover, circulating AFABP concentration was associated with ischemic stroke in cross-sectional studies, and high AFABP concentration was consistently shown to be predictive of poor functional outcome, as well as short- and long-term mortality in patients who suffered from ischemic stroke (62, 65–67).

Mechanistically, genetic ablation of AFABP in mice was recently found to protect them from severe cerebral ischemic injury induced by surgical occlusion of their middle cerebral artery, which translated to less neurological deficits and improved survival after ischemic stroke. Both circulating and cerebral AFABP concentrations were elevated in response to cerebral ischemia. The increase in AFABP, derived from microglia and infiltrating macrophages, enhanced the production of matrix metalloproteinases-9 (MMP-9) through JNK activity, which degraded the tight junction proteins in the blood brain barrier, leading to cerebral edema, increased neuro-inflammation and poor neurological outcomes (68).

## AFABP, Heart Failure, and Cardiovascular Mortality

AFABP plays a critical role in the development of heart failure and predisposes to increased cardiovascular mortality. *In vitro* studies demonstrated that adipocyte-derived AFABP possessed a negative inotropic effect on rat cardiomyocytes and could inhibit their contraction (69). In humans, circulating AFABP concentration positively correlated with circulating levels of N-terminal fragment of pro-B-type natriuretic peptide (NT-proBNP), an established marker of heart failure (70). Moreover, high circulating AFABP concentration was associated with the presence of left ventricular systolic and/or diastolic dysfunction (71–73), as well as increasing severity of clinical heart failure (74). In the Cardiovascular Health Study, circulating AFABP concentration was also shown to be a modest but independent predictor of incident heart failure among older individuals (75).

The negative impact of AFABP on cardiovascular outcomes could also be attributed to their effects on endothelial dysfunction and oxidative stress. Genetic ablation of AFABP protected mice from cardiac dysfunction secondary to diabetes and myocardial ischemia/reperfusion (MI/R) injury. AFABP, whose expression was upregulated in cardiac endothelial cells in response to acute MI/R injury and hyperglycemia, reduced phosphorylation of endothelial nitric oxide synthase (eNOS) in acute MI/R injury, and increased superoxide anions in diabetes. In both situations, endothelial dysfunction ensued, which induced oxidative stress and cardiac inflammation, leading to cardiac hypertrophy, fibrosis and impaired myocardial contractility (52). Indeed, in keeping with findings from studies in mice, high circulating AFABP concentration was associated with both short- and long-term cardiovascular morbidity and mortality in patients with established CHD (76–78),and was an independent predictor of cardiovascular deaths in patients with type 2 diabetes (79, 80).

## AFABP and Other Obesity-Related Conditions With Increased Cardiovascular Risk

AFABP is also implicated in the pathogenesis of several obesity-related complications with increased cardiovascular risk, such as NAFLD, obstructive sleep apnea (OSA) and chronic kidney disease (CKD) (90–92). In NAFLD, for instance, over-expression of AFABP in Kupffer cells of the liver induced non-alcoholic steatohepatitis in mice, while obesity-induced liver injury was alleviated by pharmacological inhibition of AFABP (93). Similar findings had been observed in humans, where circulating AFABP concentration was associated with increasing lobular inflammation, hepatocyte ballooning and higher stages of hepatic fibrosis on liver histology (94). On the other hand, elevated serum AFABP concentration was also found in patients with severe OSA compared with those with milder disease (95, 96), and the use of continuous positive airway pressure was shown to reduce circulating AFABP concentrations in a recent randomized controlled study (97). Moreover, circulating AFABP was associated with adverse renal outcomes including renal deaths in patients with type 2 diabetes (98), which could possibly be a result of macrophage infiltration in the glomerulus and interstitium, ectopic expression of AFABP in the glomerulus, as well as AFABP induced increased ER stress in the mesangial cells (99–101). Importantly, high circulating AFABP concentration was also an independent predictor of cardiovascular death in patients with end-stage renal disease (102).

## AFABP as a Therapeutic Target for CVD

Preclinical studies have demonstrated that there is great potential in targeting AFABP as a therapeutic strategy to combat CVD and its risk factors. Several AFABP inhibitors have been developed, including a few biphenyl azole, indole- and carbazole-based compounds. In particular, BMS309403 (BMS) is a selective, high-affinity small molecule oral inhibitor of AFABP which impedes the ligation of fatty acid to its binding cavity on AFABP (103). Pharmacological inhibition of AFABP using BMS alleviated endothelial dysfunction and atherosclerosis in mice with ApoE deficiency. This was accompanied by reduced cholesterol ester accumulation in macrophages, as well as attenuated expression of pro-inflammatory cytokines including MCP1, IL-6 and TNFα (104, 105). Recently, BMS was also shown to improve stroke outcomes by ameliorating neurological deficits and improving the survival in mice with cerebral ischemic injury after surgical occlusion of their middle cerebral artery (68). Moreover, BMS attenuated non-alcoholic steatohepatitis (93), improved glucose tolerance (105) and decreased toxic lipid-induced ER stress associated inflammation in the skeletal muscle of mice with dietary obesity (106). Another small molecule inhibitor HTS01037, which acts as a competitive antagonist of AFABP mediated protein-protein interactions (107), was shown to alleviate macrophage inflammation and ER stress through upregulating uncoupling protein 2 (UCP2) expression (108). In addition to these oral compounds, alternative approaches of AFABP inhibition have also been investigated. The use of neutralizing antibodies against AFABP was demonstrated to significantly reduce adipose tissue inflammation (34), hepatic glucose production (25), and whole-body insulin resistance in obese mice (109). Likewise, adipocyte targeted silencing of AFABP using short-hairpin RNA treatment resulted in significant weight reduction, improved insulin sensitivity and glycemia in obese mice (110).

Although clinical studies of both BMS and neutralizing antibodies are still not available, several compounds have been found to modulate circulating AFABP concentrations. Treatment with chloroquine in mice diminished AFABP secretion from adipocytes, resulting in a lower circulating concentration (26). In humans, atorvastatin (111), sitagliptin (112), omega-3 fatty acids (113), and angiotensin II receptor blockers (ARBs) including candesartan, olmesartan, telmisartan and valsartan (114) decreased, whereas pioglitazone (115) and canagliflozin increased circulating AFABP concentrations (116). While omega-3 fatty acids and pioglitazone directly affect AFABP expression in adipocytes, it was postulated that ARBs suppressed and canagliflozin promoted catecholamines-induced lipolysis, respectively, causing the changes in the circulating AFABP concentrations despite neutral, if not favorable effects of ARB and sodium glucose co-transporter 2 inhibitors on adiposity (114, 116).

## Conclusion

**Figure 2:**
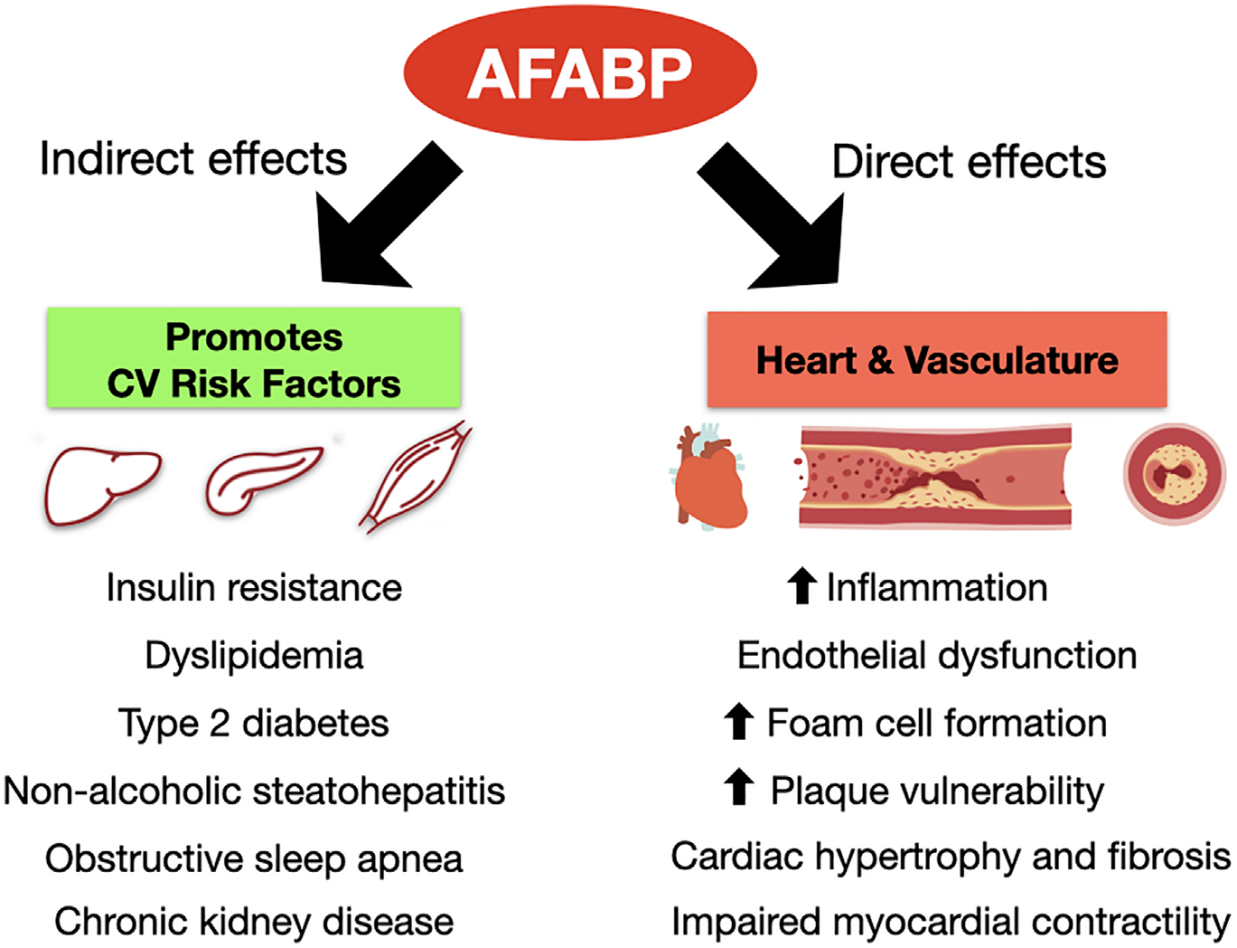
Direct and indirect effects of AFABP to the development of cardiovascular diseases. AFABP, adipocyte fatty acid-binding protein; CV, cardiovascular.

Obesity has reached pandemic levels, and so has CVD. Adipose tissue inflammation with dysregulated adipokine secretion is crucial to the pathogenesis of adverse cardiovascular outcomes in obesity. Recent mechanistic and epidemiological studies have provided further insights to support AFABP as a key player mediating this adipose-vascular cross-talk *via* direct and indirect effects (**Figure 2**). However, from a clinical perspective, further validation studies are certainly required to investigate the potential of employing AFABP as a promising marker of CVD and cardiovascular mortality for clinical application. Moreover, standardization of commercial AFABP ELISA assays is also equally important. On the other hand, while preclinical studies have clearly demonstrated AFABP as an attractive therapeutic target in battling against CVD, intervention studies to evaluate the efficacy and safety of pharmacological inhibitors of AFABP and/or neutralizing antibodies in humans are eagerly awaited. In summary, although it may still be a long way before its clinical application as a biomarker or therapeutic target, research in recent years have clearly shown that AFABP is another major adipokine linking obesity with inflammation and adverse cardiovascular outcomes.

## Author Contributions

C-HL researched the data and wrote the manuscript. DL and KL critically reviewed and edited the manuscript. KL initiated and conceptualized this review and is the guarantor of this work. All authors contributed to the article and approved the submitted version.

## Conflict of Interest

The authors declare that the research was conducted in the absence of any commercial or financial relationships that could be construed as a potential conflict of interest.

## References

[1] World Health Organization. Obesity and overweight. (2018). https://www.who.int/en/news-room/fact-sheets/detail/obesity-and-overweight.

[2] Collaboration NCDRF. Trends in adult body-mass index in 200 countries from 1975 to 2014: a pooled analysis of 1698 population-based measurement studies with 19.2 million participants. Lancet (2016) 387(10026):1377–96. 10.1016/S0140-6736(16)30054-X PMC7615134 27115820

[3] Wat NM Lam TH Janus ED Lam KS. Central obesity predicts the worsening of glycemia in southern Chinese. Int J Obes Relat Metab Disord (2001) 25(12):1789–93. 10.1038/sj.ijo.0801834 11781759

[4] Cheung BM Wat NM Man YB Tam S Thomas GN Leung GM. Development of diabetes in Chinese with the metabolic syndrome: a 6-year prospective study. Diabetes Care (2007) 30(6):1430–6. 10.2337/dc06-1820 17337491

[5] Chalasani N Younossi Z Lavine JE Charlton M Cusi K Rinella M. The diagnosis and management of nonalcoholic fatty liver disease: Practice guidance from the American Association for the Study of Liver Diseases. Hepatology (2018) 67(1):328–57. 10.1002/hep.29367 28714183

[6] Scherer PE Hill JA. Obesity, Diabetes, and Cardiovascular Diseases: A Compendium. Circ Res (2016) 118(11):1703–5. 10.1161/CIRCRESAHA.116.308999 PMC4888905 27230636

[7] Lee CH Woo YC Wang Y Yeung CY Xu A Lam KS. Obesity, adipokines and cancer: an update. Clin Endocrinol (Oxf) (2015) 83(2):147–56. 10.1111/cen.12667 25393563

[8] Collaborators GBDRF. Global, regional, and national comparative risk assessment of 84 behavioural, environmental and occupational, and metabolic risks or clusters of risks for 195 countries and territories, 1990-2017: a systematic analysis for the Global Burden of Disease Study 2017. Lancet (2018) 392(10159):1923–94. 10.1016/S0140-6736(18)32225-6 PMC6227755 30496105

[9] Collaborators GBDO Afshin A Forouzanfar MH Reitsma MB Sur P Estep K. Health Effects of Overweight and Obesity in 195 Countries over 25 Years. N Engl J Med (2017) 377(1):13–27. 10.1056/NEJMoa1614362 28604169 PMC5477817

[10] Larsson SC Back M Rees JMB Mason AM Burgess S. Body mass index and body composition in relation to 14 cardiovascular conditions in UK Biobank: a Mendelian randomization study. Eur Heart J (2020) 41(2):221–6. 10.1093/eurheartj/ehz388 PMC6945523 31195408

[11] Dale CE Fatemifar G Palmer TM White J Prieto-Merino D Zabaneh D. Causal Associations of Adiposity and Body Fat Distribution With Coronary Heart Disease, Stroke Subtypes, and Type 2 Diabetes Mellitus: A Mendelian Randomization Analysis. Circulation (2017) 135(24):2373–88. 10.1161/CIRCULATIONAHA.116.026560 PMC5515354 28500271

[12] Hansson GK. Inflammation, atherosclerosis, and coronary artery disease. N Engl J Med (2005) 352(16):1685–95. 10.1056/NEJMra043430 15843671

[13] Ridker PM. Clinical application of C-reactive protein for cardiovascular disease detection and prevention. Circulation (2003) 107(3):363–9. 10.1161/01.cir.0000053730.47739.3c 12551853

[14] Valgimigli M Ceconi C Malagutti P Merli E Soukhomovskaia O Francolini G. Tumor necrosis factor-alpha receptor 1 is a major predictor of mortality and new-onset heart failure in patients with acute myocardial infarction: the Cytokine-Activation and Long-Term Prognosis in Myocardial Infarction (C-ALPHA) study. Circulation (2005) 111(7):863–70. 10.1161/01.CIR.0000155614.35441.69 15699251

[15] Ridker PM Everett BM Thuren T MacFadyen JG Chang WH Ballantyne C. Antiinflammatory Therapy with Canakinumab for Atherosclerotic Disease. N Engl J Med (2017) 377(12):1119–31. 10.1056/NEJMoa1707914 28845751

[16] Zhang X Xu A Chung SK Cresser JH Sweeney G Wong RL. Selective inactivation of c-Jun NH2-terminal kinase in adipose tissue protects against diet-induced obesity and improves insulin sensitivity in both liver and skeletal muscle in mice. Diabetes (2011) 60(2):486–95. 10.2337/db10-0650 PMC3028348 21270260

[17] Baxa CA Sha RS Buelt MK Smith AJ Matarese V Chinander LL. Human adipocyte lipid-binding protein: purification of the protein and cloning of its complementary DNA. Biochemistry (1989) 28(22):8683–90. 10.1021/bi00448a003 2481498

[18] Coe NR Bernlohr DA. Physiological properties and functions of intracellular fatty acid-binding proteins. Biochim Biophys Acta (1998) 1391(3):287–306. 10.1016/s0005-2760(97)00205-1 9555061

[19] Shen WJ Liang Y Hong R Patel S Natu V Sridhar K. Characterization of the functional interaction of adipocyte lipid-binding protein with hormone-sensitive lipase. J Biol Chem (2001) 276(52):49443–8. 10.1074/jbc.M104095200 11682468

[20] Hofer P Boeszoermenyi A Jaeger D Feiler U Arthanari H Mayer N. Fatty Acid-binding Proteins Interact with Comparative Gene Identification-58 Linking Lipolysis with Lipid Ligand Shuttling. J Biol Chem (2015) 290(30):18438–53. 10.1074/jbc.M114.628958 PMC4513104 25953897

[21] Amri EZ Bertrand B Ailhaud G Grimaldi P. Regulation of adipose cell differentiation. I. Fatty acids are inducers of the aP2 gene expression. J Lipid Res (1991) 32(9):1449–56. 10.1016/S0022-2275(20)41912-1 1753215

[22] Cook JS Lucas JJ Sibley E Bolanowski MA Christy RJ Kelly TJ. Expression of the differentiation-induced gene for fatty acid-binding protein is activated by glucocorticoid and cAMP. Proc Natl Acad Sci U S A (1988) 85(9):2949–53. 10.1073/pnas.85.9.2949 PMC280120 2452440

[23] Kletzien RF Foellmi LA Harris PK Wyse BM Clarke SD. Adipocyte fatty acid-binding protein: regulation of gene expression in vivo and in vitro by an insulin-sensitizing agent. Mol Pharmacol (1992) 42(4):558–62. 1435736

[24] Xu A Wang Y Xu JY Stejskal D Tam S Zhang J. Adipocyte fatty acid-binding protein is a plasma biomarker closely associated with obesity and metabolic syndrome. Clin Chem (2006) 52(3):405–13. 10.1373/clinchem.2005.062463 16423904

[25] Cao H Sekiya M Ertunc ME Burak MF Mayers JR White A. Adipocyte lipid chaperone AP2 is a secreted adipokine regulating hepatic glucose production. Cell Metab (2013) 17(5):768–78. 10.1016/j.cmet.2013.04.012 PMC3755450 23663740

[26] Villeneuve J Bassaganyas L Lepreux S Chiritoiu M Costet P Ripoche J. Unconventional secretion of FABP4 by endosomes and secretory lysosomes. J Cell Biol (2018) 217(2):649–65. 10.1083/jcb.201705047 PMC5800802 29212659

[27] Josephrajan A Hertzel AV Bohm EK McBurney MW Imai SI Mashek DG. Unconventional Secretion of Adipocyte Fatty Acid Binding Protein 4 Is Mediated By Autophagic Proteins in a Sirtuin-1-Dependent Manner. Diabetes (2019) 68(9):1767–77. 10.2337/db18-1367 PMC6702637 31171562

[28] Makowski L Boord JB Maeda K Babaev VR Uysal KT Morgan MA. Lack of macrophage fatty-acid-binding protein aP2 protects mice deficient in apolipoprotein E against atherosclerosis. Nat Med (2001) 7(6):699–705. 10.1038/89076 11385507 PMC4027052

[29] Elmasri H Karaaslan C Teper Y Ghelfi E Weng M Ince TA. Fatty acid binding protein 4 is a target of VEGF and a regulator of cell proliferation in endothelial cells. FASEB J (2009) 23(11):3865–73. 10.1096/fj.09-134882 PMC2775007 19625659

[30] Laurencikiene J Skurk T Kulyte A Heden P Astrom G Sjolin E. Regulation of lipolysis in small and large fat cells of the same subject. J Clin Endocrinol Metab (2011) 96(12):E2045–9. 10.1210/jc.2011-1702 21994963

[31] Montgomery MK De Nardo W Watt MJ. Impact of Lipotoxicity on Tissue “Cross Talk” and Metabolic Regulation. Physiol (Bethesda) (2019) 34(2):134–49. 10.1152/physiol.00037.2018 30724128

[32] Wu LE Samocha-Bonet D Whitworth PT Fazakerley DJ Turner N Biden TJ. Identification of fatty acid binding protein 4 as an adipokine that regulates insulin secretion during obesity. Mol Metab (2014) 3(4):465–73. 10.1016/j.molmet.2014.02.005 PMC4060222 24944906

[33] Erbay E Babaev VR Mayers JR Makowski L Charles KN Snitow ME. Reducing endoplasmic reticulum stress through a macrophage lipid chaperone alleviates atherosclerosis. Nat Med (2009) 15(12):1383–91. 10.1038/nm.2067 PMC2790330 19966778

[34] Miao X Wang Y Wang W Lv X Wang M Yin H. The mAb against adipocyte fatty acid-binding protein 2E4 attenuates the inflammation in the mouse model of high-fat diet-induced obesity via toll-like receptor 4 pathway. Mol Cell Endocrinol (2015) 403:1–9. 10.1016/j.mce.2014.12.017 25596549

[35] Shimobayashi M Albert V Woelnerhanssen B Frei IC Weissenberger D Meyer-Gerspach AC. Insulin resistance causes inflammation in adipose tissue. J Clin Invest (2018) 128(4):1538–50. 10.1172/JCI96139 PMC5873875 29528335

[36] Weisberg SP McCann D Desai M Rosenbaum M Leibel RL Ferrante AW Jr. Obesity is associated with macrophage accumulation in adipose tissue. J Clin Invest (2003) 112(12):1796–808. 10.1172/JCI19246 PMC296995 14679176

[37] Lumeng CN Bodzin JL Saltiel AR. Obesity induces a phenotypic switch in adipose tissue macrophage polarization. J Clin Invest (2007) 117(1):175–84. 10.1172/JCI29881 PMC1716210 17200717

[38] Kratz M Coats BR Hisert KB Hagman D Mutskov V Peris E. Metabolic dysfunction drives a mechanistically distinct proinflammatory phenotype in adipose tissue macrophages. Cell Metab (2014) 20(4):614–25. 10.1016/j.cmet.2014.08.010 PMC4192131 25242226

[39] McLaughlin T Ackerman SE Shen L Engleman E. Role of innate and adaptive immunity in obesity-associated metabolic disease. J Clin Invest (2017) 127(1):5–13. 10.1172/JCI88876 28045397 PMC5199693

[40] Abdelwahab SA Owada Y Kitanaka N Adida A Sakagami H Ono M. Enhanced expression of adipocyte-type fatty acid binding protein in murine lymphocytes in response to dexamethasone treatment. Mol Cell Biochem (2007) 299(1-2):99–107. 10.1007/s11010-005-9050-1 17111194

[41] Rolph MS Young TR Shum BO Gorgun CZ Schmitz-Peiffer C Ramshaw IA. Regulation of dendritic cell function and T cell priming by the fatty acid-binding protein AP2. J Immunol (2006) 177(11):7794–801. 10.4049/jimmunol.177.11.7794 17114450

[42] Hui X Li H Zhou Z Lam KS Xiao Y Wu D. Adipocyte fatty acid-binding protein modulates inflammatory responses in macrophages through a positive feedback loop involving c-Jun NH2-terminal kinases and activator protein-1. J Biol Chem (2010) 285(14):10273–80. 10.1074/jbc.M109.097907 PMC2856232 20145251

[43] Pan Y Tian T Park CO Lofftus SY Mei S Liu X. Survival of tissue-resident memory T cells requires exogenous lipid uptake and metabolism. Nature (2017) 543(7644):252–6. 10.1038/nature21379 PMC5509051 28219080

[44] Hu B Li Y Gao L Guo Y Zhang Y Chai X. Hepatic Induction of Fatty Acid Binding Protein 4 Plays a Pathogenic Role in Sepsis in Mice. Am J Pathol (2017) 187(5):1059–67. 10.1016/j.ajpath.2017.01.002 PMC5417005 28279656

[45] Dou HX Wang T Su HX Gao DD Xu YC Li YX. Exogenous FABP4 interferes with differentiation, promotes lipolysis and inflammation in adipocytes. Endocrine (2020) 67(3):587–96. 10.1007/s12020-019-02157-8 31845180

[46] Lee CH Lam KS. Obesity-induced insulin resistance and macrophage infiltration of the adipose tissue: A vicious cycle. J Diabetes Investig (2019) 10(1):29–31. 10.1111/jdi.12918 PMC6319486 30144345

[47] Hotamisligil GS Johnson RS Distel RJ Ellis R Papaioannou VE Spiegelman BM. Uncoupling of obesity from insulin resistance through a targeted mutation in aP2, the adipocyte fatty acid binding protein. Science (1996) 274(5291):1377–9. 10.1126/science.274.5291.1377 8910278

[48] Uysal KT Scheja L Wiesbrock SM Bonner-Weir S Hotamisligil GS. Improved glucose and lipid metabolism in genetically obese mice lacking aP2. Endocrinology (2000) 141(9):3388–96. 10.1210/endo.141.9.7637 10965911

[49] Baar RA Dingfelder CS Smith LA Bernlohr DA Wu C Lange AJ. Investigation of in vivo fatty acid metabolism in AFABP/aP2(-/-) mice. Am J Physiol Endocrinol Metab (2005) 288(1):E187–93. 10.1152/ajpendo.00256.2004 15367400

[50] Tso AW Xu A Sham PC Wat NM Wang Y Fong CH. Serum adipocyte fatty acid binding protein as a new biomarker predicting the development of type 2 diabetes: a 10-year prospective study in a Chinese cohort. Diabetes Care (2007) 30(10):2667–72. 10.2337/dc07-0413 17620449

[51] Xu A Tso AW Cheung BM Wang Y Wat NM Fong CH. Circulating adipocyte-fatty acid binding protein levels predict the development of the metabolic syndrome: a 5-year prospective study. Circulation (2007) 115(12):1537–43. 10.1161/CIRCULATIONAHA.106.647503 17389279

[52] Zhou M Bao Y Li H Pan Y Shu L Xia Z. Deficiency of adipocyte fatty-acid-binding protein alleviates myocardial ischaemia/reperfusion injury and diabetes-induced cardiac dysfunction. Clin Sci (Lond) (2015) 129(7):547–59. 10.1042/CS20150073 26186740

[53] Fu Y Luo N Lopes-Virella MF Garvey WT. The adipocyte lipid binding protein (ALBP/aP2) gene facilitates foam cell formation in human THP-1 macrophages. Atherosclerosis (2002) 165(2):259–69. 10.1016/s0021-9150(02)00305-2 12417276

[54] Fu Y Luo L Luo N Garvey WT. Lipid metabolism mediated by adipocyte lipid binding protein (ALBP/aP2) gene expression in human THP-1 macrophages. Atherosclerosis (2006) 188(1):102–11. 10.1016/j.atherosclerosis.2005.10.041 16313911

[55] Zhang Y Rao E Zeng J Hao J Sun Y Liu S. Adipose Fatty Acid Binding Protein Promotes Saturated Fatty Acid-induced Macrophage Cell Death through Enhancing Ceramide Production. J Immunol (2017) 198(198):798–807. 10.4049/jimmunol.1601403 27920274 PMC5225136

[56] Kwok KHM Cheng KKY Hoo RLC Ye D Xu A Lam KSL. Adipose-specific inactivation of JNK alleviates atherosclerosis in apoE-deficient mice. Clin Sci (Lond) (2016) 130(22):2087–100. 10.1042/CS20160465 27512097

[57] Chow WS Tso AW Xu A Yuen MM Fong CH Lam TH. Elevated circulating adipocyte-fatty acid binding protein levels predict incident cardiovascular events in a community-based cohort: a 12-year prospective study. J Am Heart Assoc (2013) 2(1):e004176. 10.1161/JAHA.112.004176 23525430 PMC3603238

[58] Bagheri R Qasim AN Mehta NN Terembula K Kapoor S Braunstein S. Relation of plasma fatty acid binding proteins 4 and 5 with the metabolic syndrome, inflammation and coronary calcium in patients with type-2 diabetes mellitus. Am J Cardiol (2010) 106(8):1118–23. 10.1016/j.amjcard.2010.06.028 PMC3108486 20920650

[59] Miyoshi T Onoue G Hirohata A Hirohata S Usui S Hina K. Serum adipocyte fatty acid-binding protein is independently associated with coronary atherosclerotic burden measured by intravascular ultrasound. Atherosclerosis (2010) 211(1):164–9. 10.1016/j.atherosclerosis.2010.01.032 20193950

[60] Furuhashi M Fuseya T Murata M Hoshina K Ishimura S Mita T. Local Production of Fatty Acid-Binding Protein 4 in Epicardial/Perivascular Fat and Macrophages Is Linked to Coronary Atherosclerosis. Arterioscler Thromb Vasc Biol (2016) 36(5):825–34. 10.1161/ATVBAHA.116.307225 27013610

[61] Yeung DC Xu A Cheung CW Wat NM Yau MH Fong CH. Serum adipocyte fatty acid-binding protein levels were independently associated with carotid atherosclerosis. Arterioscler Thromb Vasc Biol (2007) 27(8):1796–802. 10.1161/ATVBAHA.107.146274 17510463

[62] Holm S Ueland T Dahl TB Michelsen AE Skjelland M Russell D. Fatty Acid binding protein 4 is associated with carotid atherosclerosis and outcome in patients with acute ischemic stroke. PLoS One (2011) 6(12):e28785. 10.1371/journal.pone.0028785 22174896 PMC3235157

[63] Agardh HE Folkersen L Ekstrand J Marcus D Swedenborg J Hedin U. Expression of fatty acid-binding protein 4/aP2 is correlated with plaque instability in carotid atherosclerosis. J Intern Med (2011) 269(2):200–10. 10.1111/j.1365-2796.2010.02304.x 21073559

[64] Peeters W de Kleijn DP Vink A van de Weg S Schoneveld AH Sze SK. Adipocyte fatty acid binding protein in atherosclerotic plaques is associated with local vulnerability and is predictive for the occurrence of adverse cardiovascular events. Eur Heart J (2011) 32(14):1758–68. 10.1093/eurheartj/ehq387 21059735

[65] Tso AW Lam TK Xu A Yiu KH Tse HF Li LS. Serum adipocyte fatty acid-binding protein associated with ischemic stroke and early death. Neurology (2011) 76(23):1968–75. 10.1212/WNL.0b013e31821e54b3 21562251

[66] Tu WJ Zeng XW Deng A Zhao SJ Luo DZ Ma GZ. Circulating FABP4 (Fatty Acid-Binding Protein 4) Is a Novel Prognostic Biomarker in Patients With Acute Ischemic Stroke. Stroke (2017) 48(6):1531–8. 10.1161/STROKEAHA.117.017128 28487339

[67] Li S Bi P Zhao W Lian Y Zhu H Xu D. Prognostic Utility of Fatty Acid-Binding Protein 4 in Patients with Type 2 Diabetes and Acute Ischemic Stroke. Neurotox Res (2018) 33(2):309–15. 10.1007/s12640-017-9792-z 28801883

[68] Liao B Geng L Zhang F Shu L Wei L Yeung PKK. Adipocyte fatty acid-binding protein exacerbates cerebral ischaemia injury by disrupting the blood-brain barrier. Eur Heart J (2020) 41:3169–80. 10.1093/eurheartj/ehaa207 PMC7556749 32350521

[69] Lamounier-Zepter V Look C Alvarez J Christ T Ravens U Schunck WH. Adipocyte fatty acid-binding protein suppresses cardiomyocyte contraction: a new link between obesity and heart disease. Circ Res (2009) 105(4):326–34. 10.1161/CIRCRESAHA.109.200501 19608978

[70] Cabre A Valdovinos P Lazaro I Bonet G Bardaji A Masana L. Parallel evolution of circulating FABP4 and NT-proBNP in heart failure patients. Cardiovasc Diabetol (2013) 12:72. 10.1186/1475-2840-12-72 23642261 PMC3653725

[71] Engeli S Utz W Haufe S Lamounier-Zepter V Pofahl M Traber J. Fatty acid binding protein 4 predicts left ventricular mass and longitudinal function in overweight and obese women. Heart (2013) 99(13):944–8. 10.1136/heartjnl-2013-303735 23598540

[72] Baessler A Lamounier-Zepter V Fenk S Strack C Lahmann C Loew T. Adipocyte fatty acid-binding protein levels are associated with left ventricular diastolic dysfunction in morbidly obese subjects. Nutr Diabetes (2014) 4:e106. 10.1038/nutd.2014.3 24513579 PMC3940827

[73] Fuseya T Furuhashi M Yuda S Muranaka A Kawamukai M Mita T. Elevation of circulating fatty acid-binding protein 4 is independently associated with left ventricular diastolic dysfunction in a general population. Cardiovasc Diabetol (2014) 13:126. 10.1186/s12933-014-0126-7 25142635 PMC4148010

[74] Liu M Zhou M Bao Y Xu Z Li H Zhang H. Circulating adipocyte fatty acid-binding protein levels are independently associated with heart failure. Clin Sci (Lond) (2013) 124(2):115–22. 10.1042/CS20120004 23013043

[75] Djousse L Bartz TM Ix JH Kochar J Kizer JR Gottdiener JS. Fatty acid-binding protein 4 and incident heart failure: the Cardiovascular Health Study. Eur J Heart Fail (2013) 15(4):394–9. 10.1093/eurjhf/hfs196 PMC3707430 23223158

[76] von Eynatten M Breitling LP Roos M Baumann M Rothenbacher D Brenner H. Circulating adipocyte fatty acid-binding protein levels and cardiovascular morbidity and mortality in patients with coronary heart disease: a 10-year prospective study. Arterioscler Thromb Vasc Biol (2012) 32(9):2327–35. 10.1161/ATVBAHA.112.248609 22679309

[77] Reiser H Klingenberg R Hof D Cooksley-Decasper S Fuchs N Akhmedov A. Circulating FABP4 is a prognostic biomarker in patients with acute coronary syndrome but not in asymptomatic individuals. Arterioscler Thromb Vasc Biol (2015) 35(8):1872–9. 10.1161/ATVBAHA.115.305365 26069234

[78] Wong YK Cheung CYY Tang CS Au KW Hai JSH Lee CH. Age-Biomarkers-Clinical Risk Factors for Prediction of Cardiovascular Events in Patients With Coronary Artery Disease. Arterioscler Thromb Vasc Biol (2018) 38(10):2519–27. 10.1161/ATVBAHA.118.311726 30354221

[79] Liu G Ding M Chiuve SE Rimm EB Franks PW Meigs JB. Plasma Levels of Fatty Acid-Binding Protein 4, Retinol-Binding Protein 4, High-Molecular-Weight Adiponectin, and Cardiovascular Mortality Among Men With Type 2 Diabetes: A 22-Year Prospective Study. Arterioscler Thromb Vasc Biol (2016) 36(11):2259–67. 10.1161/ATVBAHA.116.308320 PMC5083188 27609367

[80] Lee CH Cheung CYY Woo YC Lui DTW Yuen MMA Fong CHY. Circulating Adipocyte Fatty Acid-Binding Protein Concentrations Predict Multiple Mortality Outcomes among Men and Women with Diabetes. Clin Chem (2018) 64(10):1496–504. 10.1373/clinchem.2018.289157 30021923

[81] Boord JB Maeda K Makowski L Babaev VR Fazio S Linton MF. Adipocyte fatty acid-binding protein, aP2, alters late atherosclerotic lesion formation in severe hypercholesterolemia. Arterioscler Thromb Vasc Biol (2002) 22(10):1686–91. 10.1161/01.atv.0000033090.81345.e6 PMC4027051 12377750

[82] Fu Y Luo N Lopes-Virella MF. Oxidized LDL induces the expression of ALBP/aP2 mRNA and protein in human THP-1 macrophages. J Lipid Res (2000) 41(12):2017–23. 10.1016/S0022-2275(20)32363-4 11108735

[83] Kazemi MR McDonald CM Shigenaga JK Grunfeld C Feingold KR. Adipocyte fatty acid-binding protein expression and lipid accumulation are increased during activation of murine macrophages by toll-like receptor agonists. Arterioscler Thromb Vasc Biol (2005) 25(6):1220–4. 10.1161/01.ATV.0000159163.52632.1b 15705927

[84] Weinbrenner T Schroder H Escurriol V Fito M Elosua R Vila J. Circulating oxidized LDL is associated with increased waist circumference independent of body mass index in men and women. Am J Clin Nutr (2006) 83(1):30–5; quiz 181-2. 10.1093/ajcn/83.1.30 16400046

[85] Moludi J Maleki V Jafari-Vayghyan H Vaghef-Mehrabany E Alizadeh M. Metabolic endotoxemia and cardiovascular disease: A systematic review about potential roles of prebiotics and probiotics. Clin Exp Pharmacol Physiol (2020) 47(6):927–39. 10.1111/1440-1681.13250 31894861

[86] Song J Ren P Zhang L Wang XL Chen L Shen YH. Metformin reduces lipid accumulation in macrophages by inhibiting FOXO1-mediated transcription of fatty acid-binding protein 4. Biochem Biophys Res Commun (2010) 393(1):89–94. 10.1016/j.bbrc.2010.01.086 20102700

[87] Hoo RL Shu L Cheng KK Wu X Liao B Wu D. Adipocyte Fatty Acid Binding Protein Potentiates Toxic Lipids-Induced Endoplasmic Reticulum Stress in Macrophages via Inhibition of Janus Kinase 2-dependent Autophagy. Sci Rep (2017) 7:40657. 10.1038/srep40657 28094778 PMC5240568

[88] Tuncman G Erbay E Hom X De Vivo I Campos H Rimm EB. A genetic variant at the fatty acid-binding protein aP2 locus reduces the risk for hypertriglyceridemia, type 2 diabetes, and cardiovascular disease. Proc Natl Acad Sci U S A (2006) 103(18):6970–5. 10.1073/pnas.0602178103 PMC1447594 16641093

[89] Furuhashi M Yuda S Muranaka A Kawamukai M Matsumoto M Tanaka M. Circulating Fatty Acid-Binding Protein 4 Concentration Predicts the Progression of Carotid Atherosclerosis in a General Population Without Medication. Circ J (2018) 82(4):1121–9. 10.1253/circj.CJ-17-1295 29445067

[90] Francque SM van der Graaff D Kwanten WJ. Non-alcoholic fatty liver disease and cardiovascular risk: Pathophysiological mechanisms and implications. J Hepatol (2016) 65(2):425–43. 10.1016/j.jhep.2016.04.005 27091791

[91] Tietjens JR Claman D Kezirian EJ De Marco T Mirzayan A Sadroonri B. Obstructive Sleep Apnea in Cardiovascular Disease: A Review of the Literature and Proposed Multidisciplinary Clinical Management Strategy. J Am Heart Assoc (2019) 8(1):e010440. 10.1161/JAHA.118.010440 30590966 PMC6405725

[92] Collaboration GBDCKD. Global, regional, and national burden of chronic kidney disease, 1990-2017: a systematic analysis for the Global Burden of Disease Study 2017. Lancet (2020) 395(10225):709–33. 10.1016/S0140-6736(20)30045-3 PMC7049905 32061315

[93] Hoo RL Lee IP Zhou M Wong JY Hui X Xu A. Pharmacological inhibition of adipocyte fatty acid binding protein alleviates both acute liver injury and non-alcoholic steatohepatitis in mice. J Hepatol (2013) 58(2):358–64. 10.1016/j.jhep.2012.10.022 23108115

[94] Milner KL van der Poorten D Xu A Bugianesi E Kench JG Lam KS. Adipocyte fatty acid binding protein levels relate to inflammation and fibrosis in nonalcoholic fatty liver disease. Hepatology (2009) 49(6):1926–34. 10.1002/hep.22896 19475694

[95] Lam DC Xu A Lam KS Lam B Lam JC Lui MM. Serum adipocyte-fatty acid binding protein level is elevated in severe OSA and correlates with insulin resistance. Eur Respir J (2009) 33(2):346–51. 10.1183/09031936.50075408 19181913

[96] Catala R Cabre A Hernandez-Flix S Ferre R Sangenis S Plana N. Circulating FABP4 and FABP5 levels are differently linked to OSA severity and treatment. Sleep (2013) 36(12):1831–7. 10.5665/sleep.3210 PMC3825432 24293757

[97] Lui MMS Mak JCW Chong PWC Lam DCL Ip MSM. Circulating adipocyte fatty acid-binding protein is reduced by continuous positive airway pressure treatment for obstructive sleep apnea-a randomized controlled study. Sleep Breath (2019) 24:817–24. 10.1007/s11325-019-01893-5 31372823

[98] Lee CH Cheung CYY Woo YC Lui DTW Yuen MMA Fong CHY. Prospective associations of circulating adipocyte fatty acid-binding protein levels with risks of renal outcomes and mortality in type 2 diabetes. Diabetologia (2019) 62(1):169–77. 10.1007/s00125-018-4742-8 30267180

[99] Nguyen D Ping F Mu W Hill P Atkins RC Chadban SJ. Macrophage accumulation in human progressive diabetic nephropathy. Nephrol (Carlton) (2006) 11(3):226–31. 10.1111/j.1440-1797.2006.00576.x 16756636

[100] Tanaka M Furuhashi M Okazaki Y Mita T Fuseya T Ohno K. Ectopic expression of fatty acid-binding protein 4 in the glomerulus is associated with proteinuria and renal dysfunction. Nephron Clin Pract (2014) 128(3-4):345–51. 10.1159/000368412 25592475

[101] Yao F Li Z Ehara T Yang L Wang D Feng L. Fatty Acid-Binding Protein 4 mediates apoptosis via endoplasmic reticulum stress in mesangial cells of diabetic nephropathy. Mol Cell Endocrinol (2015) 411:232–42. 10.1016/j.mce.2015.05.003 25958041

[102] Furuhashi M Ishimura S Ota H Hayashi M Nishitani T Tanaka M. Serum fatty acid-binding protein 4 is a predictor of cardiovascular events in end-stage renal disease. PLoS One (2011) 6(11):e27356. 10.1371/journal.pone.0027356 22102888 PMC3213139

[103] Furuhashi M Hotamisligil GS. Fatty acid-binding proteins: role in metabolic diseases and potential as drug targets. Nat Rev Drug Discov (2008) 7(6):489–503. 10.1038/nrd2589 18511927 PMC2821027

[104] Lee MY Li H Xiao Y Zhou Z Xu A Vanhoutte PM. Chronic administration of BMS309403 improves endothelial function in apolipoprotein E-deficient mice and in cultured human endothelial cells. Br J Pharmacol (2011) 162(7):1564–76. 10.1111/j.1476-5381.2010.01158.x PMC3057294 21175571

[105] Furuhashi M Tuncman G Gorgun CZ Makowski L Atsumi G Vaillancourt E. Treatment of diabetes and atherosclerosis by inhibiting fatty-acid-binding protein aP2. Nature (2007) 447(7147):959–65. 10.1038/nature05844 PMC4076119 17554340

[106] Bosquet A Girona J Guaita-Esteruelas S Heras M Saavedra-Garcia P Martinez-Micaelo N. FABP4 inhibitor BMS309403 decreases saturated-fatty-acid-induced endoplasmic reticulum stress-associated inflammation in skeletal muscle by reducing p38 MAPK activation. Biochim Biophys Acta Mol Cell Biol Lipids (2018) 1863(6):604–13. 10.1016/j.bbalip.2018.03.004 29550588

[107] Hertzel AV Hellberg K Reynolds JM Kruse AC Juhlmann BE Smith AJ. Identification and characterization of a small molecule inhibitor of Fatty Acid binding proteins. J Med Chem (2009) 52(19):6024–31. 10.1021/jm900720m PMC2755644 19754198

[108] Xu H Hertzel AV Steen KA Wang Q Suttles J Bernlohr DA. Uncoupling lipid metabolism from inflammation through fatty acid binding protein-dependent expression of UCP2. Mol Cell Biol (2015) 35(6):1055–65. 10.1128/MCB.01122-14 PMC4333098 25582199

[109] Burak MF Inouye KE White A Lee A Tuncman G Calay ES. Development of a therapeutic monoclonal antibody that targets secreted fatty acid-binding protein aP2 to treat type 2 diabetes. Sci Transl Med (2015) 7(319):319ra205. 10.1126/scitranslmed.aac6336 26702093

[110] Won YW Adhikary PP Lim KS Kim HJ Kim JK Kim YH. Oligopeptide complex for targeted non-viral gene delivery to adipocytes. Nat Mater (2014) 13(12):1157–64. 10.1038/nmat4092 25282508

[111] Karpisek M Stejskal D Kotolova H Kollar P Janoutova G Ochmanova R. Treatment with atorvastatin reduces serum adipocyte-fatty acid binding protein value in patients with hyperlipidaemia. Eur J Clin Invest (2007) 37(8):637–42. 10.1111/j.1365-2362.2007.01835.x 17635574

[112] Furuhashi M Hiramitsu S Mita T Fuseya T Ishimura S Omori A. Reduction of serum FABP4 level by sitagliptin, a DPP-4 inhibitor, in patients with type 2 diabetes mellitus. J Lipid Res (2015) 56(12):2372–80. 10.1194/jlr.M059469 PMC4655983 26467280

[113] Furuhashi M Hiramitsu S Mita T Omori A Fuseya T Ishimura S. Reduction of circulating FABP4 level by treatment with omega-3 fatty acid ethyl esters. Lipids Health Dis (2016) 15:5. 10.1186/s12944-016-0177-8 26754658 PMC4710044

[114] Furuhashi M Mita T Moniwa N Hoshina K Ishimura S Fuseya T. Angiotensin II receptor blockers decrease serum concentration of fatty acid-binding protein 4 in patients with hypertension. Hypertens Res (2015) 38(4):252–9. 10.1038/hr.2015.2 25672659

[115] Cabre A Lazaro I Girona J Manzanares JM Marimon F Plana N. Fatty acid binding protein 4 is increased in metabolic syndrome and with thiazolidinedione treatment in diabetic patients. Atherosclerosis (2007) 195(1):e150–8. 10.1016/j.atherosclerosis.2007.04.045 17553506

[116] Furuhashi M Matsumoto M Hiramitsu S Omori A Tanaka M Moniwa N. Possible Increase in Serum FABP4 Level Despite Adiposity Reduction by Canagliflozin, an SGLT2 Inhibitor. PLoS One (2016) 11(4):e0154482. 10.1371/journal.pone.0154482 27124282 PMC4849662

